# Combined targeting of PI3K and MEK effector pathways via CED for DIPG therapy

**DOI:** 10.1093/noajnl/vdz004

**Published:** 2019-05-28

**Authors:** Raymond Chang, Umberto Tosi, Julia Voronina, Oluwaseyi Adeuyan, Linda Y Wu, Melanie E Schweitzer, David J Pisapia, Oren J Becher, Mark M Souweidane, Uday B Maachani

**Affiliations:** 1 Department of Neurosurgery, Weill Cornell Medicine, New York, New York; 2 Department of Pathology and Laboratory Medicine, Weill Cornell Medicine, New York, New York; 3 Department of Pediatrics, Northwestern University, Chicago, Illinois; 4 Division of Hematology-Oncology and Stem Cell Transplant, Ann & Robert Lurie Children’s Hospital of Chicago, Chicago, Illinois; 5 Department of Neurosurgery, Memorial Sloan Kettering Cancer Center, New York, New York

**Keywords:** combinatorial therapy, convection-enhanced delivery, MEK, midline glioma, PI3K

## Abstract

**Background:**

Midline gliomas like diffuse intrinsic pontine glioma (DIPG) carry poor prognosis and lack effective treatment options. Studies have implicated amplifications in the phosphatidylinositol 3-kinase (PI3K) signaling pathway in tumorigenesis; compensatory activation of parallel pathways (eg, mitogen-activated protein kinase [MEK]) may underlie the resistance to PI3K inhibition observed in the clinic.

**Methods:**

Three patient-derived cell lines (SU-DIPG-IV, SU-DIPG-XIII, and SF8628) and a mouse-derived brainstem glioma cell line were treated with PI3K (ZSTK474) and MEK (trametinib) inhibitors, alone or in combination. Synergy was analyzed using Chou-Talalay combination index (CI). These agents were also used alone or in combination in a subcutaneous SU-DIPG-XIII tumor model and in an intracranial genetic mouse model of DIPG, given via convection-enhanced delivery (CED).

**Results:**

We found that these agents abrogate cell proliferation in a dose-dependent manner. Combination treatments were found to be synergistic (CI < 1) across cell lines tested. They also showed significant tumor suppression when given systemically against a subcutaneous DIPG model (alone or in combination) or when given via direct intracranial injection (CED) in a intracranial DIPG mouse model (combination only, median survival 47 vs 35 days post-induction, *P* = .038). No significant short- or long-term neurotoxicity of ZSTK474 and trametinib delivered via CED was observed.

**Conclusions:**

Our data indicate that ZSTK474 and trametinib combinatorial treatment inhibits malignant growth of DIPG cells in vitro and in vivo, prolonging survival. These results suggest a promising new combinatorial approach using CED for DIPG therapy, which warrants further investigation.

**Key Points**

1. PI3K and MEK inhibitions act synergistically against DIPG.
2. CED of ZSTK474 and trametinib is safe and beneficial against a DIPG mouse model.

**Importance of the Study**

This study demonstrates an actionable deregulation of the PI3K and MEK signaling pathways in DIPG. Targeting both pathways with ZSTK474 and trametinib, respectively, results in synergistic inhibition of cell viability. Treatment with each agent alone, albeit beneficial, resulted in upregulation of the nontargeted pathway. Novel intracranial delivery of ZSTK474 and MEK via CED, a drug delivery approach currently used in clinical trials for DIPG, resulted in a significant survival benefit in mice. Concurrently, we demonstrate that CED of these two agents resulted in no neurotoxic effects in a murine model and has potential for rapid clinical translation.

Pediatric midline gliomas, of which diffuse intrinsic pontine glioma (DIPG) is the most common variant, are universally lethal malignancies with a median survival of less than 1 year.^1^ External beam radiation is the standard of care. However, treatment only prolongs survival by a median of 3 months.^2^ This stagnating prognosis is due, in part, to the failure of most chemotherapeutics to cross the blood–brain barrier (BBB) and achieve high concentrations in the tumor.^2,3^ Local delivery strategies, such as convection-enhanced delivery (CED), in which a cannula is surgically placed in the tumor, can circumvent this problem. In a phase I clinical trial of DIPG patients, CED achieved both high regional concentrations of the therapeutic agent and minimal systemic exposure and toxicity.^4–6^

CED allows for a wide array of non-BBB-crossing therapeutics to be used. Recently, platelet-derived growth factor receptor, a receptor tyrosine kinase (RTK), has emerged as a possible target as it is estimated to be overexpressed in 80% of DIPG cases. Moreover, nearly 50% of DIPGs exhibit amplifications in the RTK/phosphatidylinositol 3-kinase (PI3K)/protein kinase B (AKT)/mammalian target of rapamycin (mTOR) signaling pathway.^7,8^ Similarly, a recent molecular meta-analysis of 1000 high pediatric glioma and DIPG samples identified common alterations in the PI3K-MAPK and mitogen-activated protein kinase (MEK) signaling pathways.^9^ Previously, we reported that inhibitors of RTKs and their downstream effectors, including dasatinib (RTK inhibitor), everolimus (mTOR inhibitor), and perifosine (PI3K/AKT inhibitor) had effective inhibition patient-derived DIPG cells growth.^10,11^

However, monotherapy using PI3K inhibitors or upstream RTK targeting has thus far proven to be insufficient in clinical trials for DIPG and other cancers.^12,13^ Besides poor BBB penetration, resistance may be due to activation of alternative signaling pathways that compensate for PI3K/AKT/mTOR inhibition and allow tumor cells to evade the antiproliferative effects of monotherapy.^14^ Combination therapy targeting multiple pathways may thus be a more effective strategy.

Co-inhibition of the RAS–ERK pathway has shown promise against glioblastoma,^15^ DIPG,^10^ and other solid cancers.^16,17^ Crosstalk between the two pathways occurs at multiple points, including the ability of ERK and the downstream kinase ribosomal S6 protein kinase to compensate for AKT in the activation of mammalian target of rapamycin complex 1 (mTORC1) via inhibitory tuberous sclerosis complex 2 phosphorylation, MEK suppression of PI3K signaling by promoting membrane localization of phosphatase and tensin homolog, and AKT suppression of RAF via phosphorylation, as schematized in Figure 1.^18^ Unfortunately, the BBB penetration of agents targeting RAS–ERK pathway and PI3K/AKT/mTOR pathway inhibitors is poor or not fully characterized and thus these agents necessitate the use of CED.^19^

**Fig. 1:**
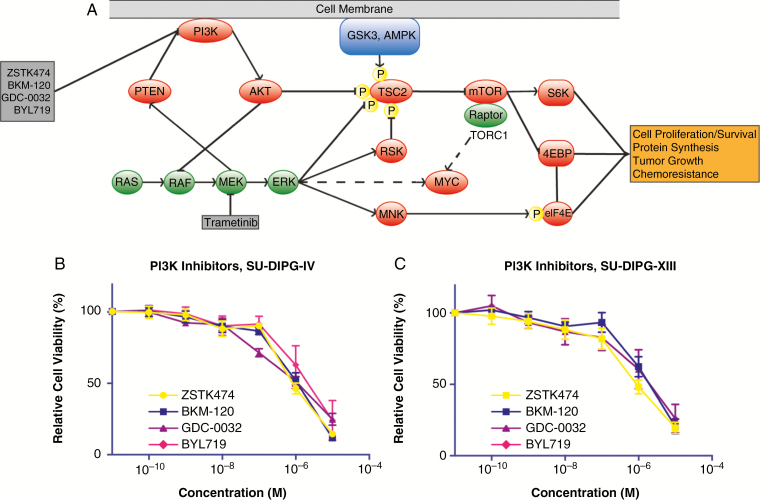
PI3K inhibition in diffuse intrinsic pontine glioma (DIPG). Schematic pathway representing the role of PI3K and mitogen-activated protein kinase (MEK) inhibition and relevant signaling molecules (A). Multiple points of compensation and feedback inhibition are a feature of the two pathways. ERK and the downstream kinase ribosomal S6 protein kinase (RSK) can compensate for AKT in the activation of mammalian target of rapamycin complex 1 (mTORC1) via inhibitory tuberous sclerosis complex 2 (TSC2) phosphorylation. Cross-inhibition occurs via MEK suppression of PI3K by promoting the membrane localization phosphatase and tensin homolog (PTEN), and AKT suppression of RAS activation via RAF inhibition. Cell viability following PI3K inhibitor treatment in SU-DIPG-IV (B) and SU-DIPG-XIII (C) cells. Errors bars SD.

In this study, we evaluate novel PI3K agents for use against DIPG and demonstrate synergistic effects by targeting both PI3K and MEK/ERK pathways. We show that ZSTK474, a PI3K inhibitor, and trametinib, a MEK inhibitor, synergistically inhibit DIPG cell proliferation in vitro and in vivo, prolonging survival in a genetically engineered mouse DIPG model following CED administration.

## Materials and Methods

### Cell Culture

Three patient-derived (SU-DIPG-IV, SU-DIPG-XIII, and SF8628) and a mouse-derived (mBSG) cell lines were used for in vitro experiments.^20,21^ SU-DIPG-IV and SU-DIPG-XIII were obtained from Dr. Michelle Monje (Stanford University School of Medicine, Stanford, CA). SF8628 cells were obtained from Dr. Rintaro Hashizume and the Gupta Laboratory (University of California San Francisco School of Medicine, San Francisco, CA). Mouse-derived brain stem glioma cells were established from the genetically engineered mouse model (GEMM) of DIPG generated by our group and using methodology previously described.^21^ Cells were grown as previously described.^10,11^

### Cell Viability Assays

Cells were seeded in 96-well plates at 5000 cells per well 24 hours prior to treatment. Cell viability was assessed 48 and 72 hours after treatment using CellTiter-Glo (Promega). Luminescence was normalized to vehicle treatment. All cell viability assays were done in triplicate for each experiment and repeated at least three times. GI_50_ values, defined as treatment dose at which cell viability was decreased to 50% of vehicle treatment condition, were estimated from log (agonist) versus response including variable slope (four parameters) statistics in GraphPad Prism. Separate cell viability assays were performed to analyze whether any of four novel PI3K agents—ZSTK474 (LC Laboratories), BKM120, GDC-0032, and BYL719 (provided by the Developmental Therapeutics Program at the National Cancer Institute)—demonstrated more potent inhibitory effects on DIPG. In addition, assays were used to determine whether combination therapy with ZSTK474 and trametinib (Developmental Therapeutics Program at the National Cancer Institute) would result in synergistic inhibition of DIPG. Each cell line was treated with each drug either individually or in combination. Combination index (CI) was calculated using Compusyn software (Combosyn) using the Chou-Talalay method.^22^

### Western Blot Analysis

Protein levels of phosphorylated and total AKT and ERK (pAKT, pERK, AKT, ERK, respectively) were quantified by immunoblotting to determine PI3K and MEK activity. Cell lines were cultured until 70% confluence, treated with ZSTK474 and trametinib individually or in combination at GI_50_ concentrations (ZSTK474 GI_50_: 860 nM for SU-DIPG-IV and 910 nM for SU-DIPG-XIII; trametinib GI_50_: 280 nM for SU-DIPG-IV and 13 nM for SU-DIPG-XIII) and harvested after 24 hours. Lysates were prepared by washing treated and nontreated cells with cold phosphate-buffered saline (PBS) followed by lysis with modified RIPA buffer (150 mM NaCl, 50 mM Tris-HCl [pH 8], 1% NP-40, 0.5% Na deoxycholate, and 0.1% SDS) supplemented with protease and phosphatase inhibitor cocktail (Cell Signaling Technology). Protein concentrations were assessed using Bradford assay (Alfa Aesar). SDS-PAGE was used to resolve lysates using 4%–20% polyacrylamide gels (Bio-Rad Laboratories). These were then transferred to PVDF membranes (Thermo Fisher) and blocked with 5% blotting-grade blocker (Bio-Rad) in TBS/0.1% Tween-20. Membranes were probed using several primary antibodies from Cell Signaling Technology: pAKT Ser473 (1:1000; #4060), pAKT-Thr308 (1:1000; #13038), pERK1/2Thr202/Tyr204 (1:1000; #4370), total ERK1/2 (1:1000; #4695), and GAPDH (1:1000; #5174). Total AKT1 (#60203-1-lg) diluted at 1:1000 was obtained from Proteintech. Appropriate secondary antibodies conjugated with horseradish peroxidase (Abcam) were incubated in blocking buffer. Western blot detection was performed with Amersham ECL kit (GE Healthcare). Each experiment was repeated independently three times. Semi-quantification of western blots was completed using ImageJ software (National Institutes of Health).

### Subcutaneous Patient-Derived Xenograft Mouse Model

All procedures conducted in mice are approved by the Weill Cornell Medicine Institutional Animal Care and Use Committee and are consistent with the recommendations of the American Veterinary Medical Association and the National Institutes of Health Guide for the Care and Use of Laboratory Animals. SU-DIPG-XIII cells were transfected with lenti-viral CAG-Luciferase (firefly), (GFP-Puro) according to manufacturer recommendations (#LVP570, GenTraget Inc.). Four-week-old females NOD.CB17-Prkdcscid/NcrCrl mice (Charles River; weight 18–23 g) were injected subcutaneously with 4.0 × 10^6^ GFP and luciferase-expressing SU-DIPG-XIII cells re-suspended in 100 μL Matrigel (BD Biosciences).

Treatment was started 2 weeks post implantation of tumor cells and confirmation of tumor establishment with bioluminescence imaging. Mice were separated into four treatment groups: ZSTK474 (*n* = 6), trametinib (*n* = 7), ZSTK474 and trametinib (combination) (*n* = 6), and vehicle (*n* = 6). Animals were treated via intraperitoneal (IP) injection once daily, for 10 days with either trametinib (3 mg/kg/day), ZSTK474 (400 mg/kg/day), or the combination of trametinib and ZSTK474 dissolved in DMSO and then diluted in PBS (without calcium and magnesium) to the desired concentration (total volume of 200 μL). The change in tumor volume was assessed with bioluminescence, measured immediately before treatment and posttreatment once weekly until day 60 after which animals were killed. Most mice showed some sign of systemic toxicity in the form of weight loss or food avoidance. No animal, however, reached euthanasia endpoints. Notably, animals with undetectable luciferase signaling at any time point were removed from two-way analysis of variance (ANOVA) analysis (*n* = 1 for each group except trametinib, *n* = 4).

### GEMM of DIPG

Survival experiments used *Nestin*-Tv-a (Ntv-a);*p53*^*fl*/*fl*^ mice. Induction of brainstem gliomas (BSGs) in mice was accomplished using DF1 virus-producing cells transfected with replication-competent avian sarcoma (RCAS) plasmids (RCAS-PDGF-B-HA, RCAS-Cre, RCAS-H3.3K27M-GFP, and RCAS-Luciferase), as previously described.^21^ Injections were performed on postnatal (P) day 3–5 after using hypothermia for anesthetizing animals on ice. Bioluminescent imaging was used to confirm initial transfection. Mice with confirmed initial transfections were separated into combination treatment (*n* = 11) and vehicle (*n* = 10) groups, monitored daily, weighed at least twice weekly and killed upon onset of brain tumor symptoms (eg, lethargy, ataxia, macrocephaly, weight loss beyond 20%). Treatments were performed at days 21 and 28 post tumor induction under isoflurane anesthesia. Animals were treated with CED of either ZSTK474 or trametinib via microinjection infusion (CED) in to brainstem at GI_90_ concentrations (1.9 μM for both drugs) in 1% DMSO in PBS, or 1% DMSO in PBS alone as control. Injection site was 1-mm posterior, 0.8-mm lateral, and 4-mm deep to lambda. A Nanofill 10 μL syringe (World Precision Instruments, Inc.) with a 32-gauge needle was used. Mice were placed in a Kopf small animal stereotactic frame. Infusion volume was 5 μL over 10 minutes.

### Bioluminescence Imaging

Mice were imaged once weekly using a Bruker In-Vivo Xtreme small animal imaging system (at The Citigroup Biomedical Imaging Center of Weill Cornell Medicine). For imaging, mice were isoflurane anesthetized (2% in oxygen) and examined for tumor bioluminescence 10 minutes following IP injection of d-luciferin (150 mg per kg, Gold Biotechnology) as per manufacturer’s protocol.

### Neurotoxicity Assessment of CED Treatments

To assess for treatment-related neurotoxicity or neuroinflammation, adult (6–8 weeks old) naïve mice were used. They were injected via CED twice as described earlier and monitored regularly following CED for difficulties with ambulation, eating, drinking, defecating, urinating, or grooming. A total of eight animals were divided into four groups —treatment with the combination of ZSTK474 and trametinib versus vehicle, and killed at 2 days versus 14 days after the last CED. Animals were treated under the same protocol as GEMM survival experiments. No animals displayed any immediate or delayed neurological deficits postoperatively.

### Histological Assessment of GEMM Experiments

Mouse brains were harvested after formalin perfusion and cryopreserved using Optimal Cutting Temperature compound (Fisher Healthcare). Sections were cut 5-μm thick using Thermo Scientific Cryotome FSE cryostats. Tumors were confirmed using hematoxylin & eosin (H&E) staining, Ki67 (D3B5) (Cell Signaling Technology, #12202), H3.3K27M (D3B5T) (Cell Signaling Technology, #74829), and luciferase (Abcam, #ab185924) staining. Neurotoxicity was assessed via H&E and immunohistochemistry for glial fibrillary acidic protein (GFAP) (Agilent, #Z033429-2), Ionized calcium binding adaptor molecule 1 (Iba1) (Abcam, #ab5076), and cluster of differentiation 3 (CD3) (Abcam, # ab16669). Staining and immunohistochemistry were performed by the Weill Cornell Pathology Core. Slides were reviewed by D.J.P., a board-certified neuropathologist, for neurohistopathological changes observed in the treatment group against vehicle-treated animals.

### Statistical Analysis

**Fig. 2:**
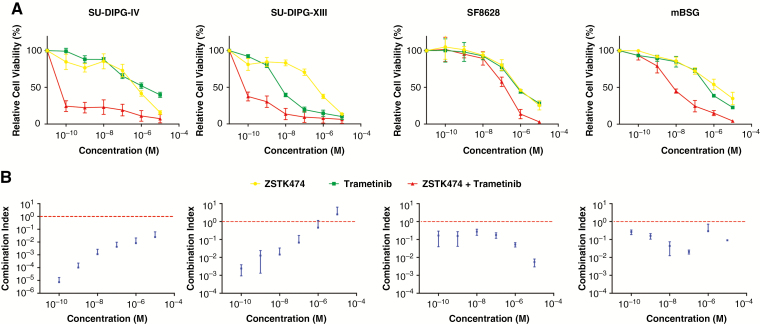
Dual inhibition of PI3K and mitogen-activated protein kinase (MEK) pathways. Cell survival studies after treatment with ZSTK474 (PI3K inhibitor), trametinib (MEK inhibitor), or their combination (A). The Chou-Talalay method for evaluating drug combinations was applied to generate a combination index (CI) at 1:1 concentration of each drug (B). CI < 1 indicates synergism. Error bars SD.

For descriptive purposes, data were represented as mean ± SD, with the exception of immunoblot semi-quantification, where SEM was used. Data were analyzed with one-way ANOVA with multiple comparisons against a control (eg, vehicle treatment), lowest GI_50_ for PI3K inhibitors (ie, ZSTK474) (Figure 2B and C) or group-by-group, performed with GraphPad Prism 7.0. Bonferroni’s and Tukey’s post hoc tests were used. For subcutaneous xenografts studies, tumor bioluminescence was quantified and analyzed by two-way ANOVA. Survival data were plotted as Kaplan–Meier curves and analyzed with the log rank (Mantel–Cox) test with GraphPad Prism 7.0. A *P* value of less than .05 was considered statistically significant.

## Results

### PI3K and MEK Inhibition Effects DIPG Cell Growth In Vitro

DIPG cells in culture were treated with various PI3K inhibitors (ZSTK474,^23^ BKM120,^24^ GDC-0032,^25^ and BYL719^26^) to determine effect on cell proliferation. Each of these agents has different cytostatic and cytotoxic effects and isoform specificity, summarized in Table 1. We observed dose- and cell line-dependent effects of PI3K inhibitors on cell viability (Figure 1B and C). There was a difference in relative cell viability only for SU-DIPG-IV when treated with selected PI3K inhibitors, as measured by estimated GI_50_ values (one-way ANOVA, *P* < .01) (Figure 1B; Table 1). ZSTK474, the agent with the lowest GI_50_, had significantly lower GI_50_ than BKM120 only (uncorrected *P* = .013) after Bonferroni correction for multiple comparisons (BYL719, *P* = .18; GDC-0032, *P* = 0.42). No difference in relative cell viability was found when treating SU-DIPG-XIII cells (Figure 1C; Table 1). As there is some evidence that specific inhibition of the α isoforms of PI3K may lead to rebound activity due to β isoforms, we decided to exclude isoform-specific PI3K inhibitors BYL179 and GDC-0032 from further experiments. Moreover, ZSTK474 was previously reported to have high oral maximal tolerated dose in mice.^27,28^ Thus, ZSTK474 was used for further experiments.

**Table 1.** Summary of GI_50_ for novel PI3K inhibitors in diffuse intrinsic pontine glioma (DIPG) cell lines, along with each agent’s specificity

	GI_50_ (SD) (μM)		Isoform Specificity
	SU-DIPG-IV	SU-DIPG-XIII	
ZSTK474	0.86 (0.15)	0.91 (0.26)	All (α, β, γ, δ)
BKM120	1.23 (0.02)	1.37 (0.18)	All (α, β, γ, δ)
GDC-0032	3.68 (1.26)	2.41 (2.19	Class I (α)
BYL719	1.02 (0.26)	0.23 (0.03)	Class I (α)

PI3K inhibitors selected from those currently in phase I or II clinical trials. Significant difference in relative cell viability was found when DIPG IV was treated with PI3K inhibitors (one-way analysis of variance, *F*(3,8) = 12.7, *P* < .01), but not DIPG XIII. Post hoc pair-wise *t* tests were carried out between ZSTK474, which had the lowest GI_50_, and other PI3K inhibitors. ZSTK474 had a significantly lower GI_50_ than only BKM120 (*P* = .013) after Bonferroni correction for multiple comparisons (BYL719, *P* = .18; GDC-0032, *P* = .42).

Combination treatment performed with ZSTK474 and trametinib, a MEK inhibitor (which was previously shown to have growth inhibitory effects on DIPG),^10^ resulted in a more potent inhibition of cell viability in patient- and mouse-derived DIPG cells compared to each drug individually (Figure 2A). Analysis using the Chou-Talalay CI showed that the abrogation in cell viability was strongly synergistic (CI < 1 synergistic, CI = 1 additive, CI > 1 antagonistic) at a wide array of concentrations and across cell lines.^22^ The only exception was treatment of SU-DIPG-XIII cells at 10 µM, the highest concentration tested (Figure 2B). The mutational burden of each cell line is summarized in Table 2, potentially explaining the differential responses to treatment with ZSTK474, trametinib, and their combination observed.

**Table 2.**
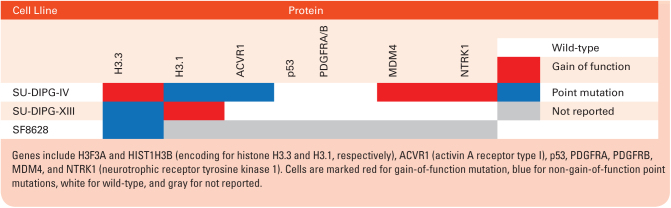
Overview of mutation status of relevant genes in cell lines used in cell viability experiments

### Downstream Effects of PI3K and MEK Inhibition on DIPG Cells In Vitro

**Fig. 3:**
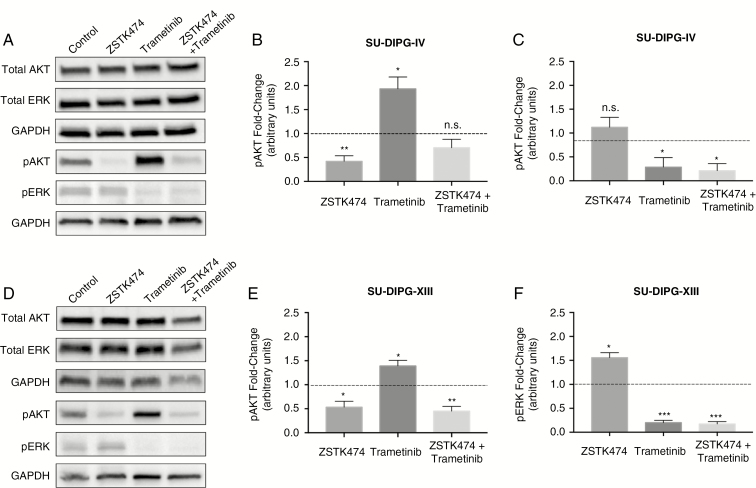
Effects of PI3K and mitogen-activated protein kinase (MEK) inhibition. Representative blots for SU-DIPG-IV cells treated with ZSTK474, trametinib, or their combination (A). Semi-quantification of pAKT (B) and pERK (C) as protein expression fold-change over 0.1% DMSO vehicle. Representative blots for SU-DIPG-XIII cells treated with ZSTK474, trametinib, or their combination (D). Semi-quantification of pAKT (E) and pERK (F) as protein expression fold-change over 0.1% DMSO vehicle. One-way *t* tests were conducted against fold-change of 1, *P < .05, **P < 0.01, ***P < .001. Error bars SEM.

Protein levels of phosphorylated and total AKT and ERK were quantified by immunoblotting to determine PI3K and MEK activity in the context of ZSTK474 and/or trametinib treatment at GI_50_ concentration (Figure 3). Treatment did not affect total AKT or ERK protein expression levels (Supplementary Figure 1A–D) in both DIPG cell lines. Treatment with ZSTK474 alone and combination with tramatinib decreased pAKT expression levels relative to vehicle (Figure 3B and E). Treatment with trametinib alone increased pAKT expression levels relative to vehicle, as expected. pERK expression levels were not affected by ZSTK474 treatment alone but were decreased after trametinib alone and both drugs in combination were given (Figure 3C and F).

### Antitumor Effects in Subcutaneous and GEMM Animal Models of DIPG

**Fig. 4:**
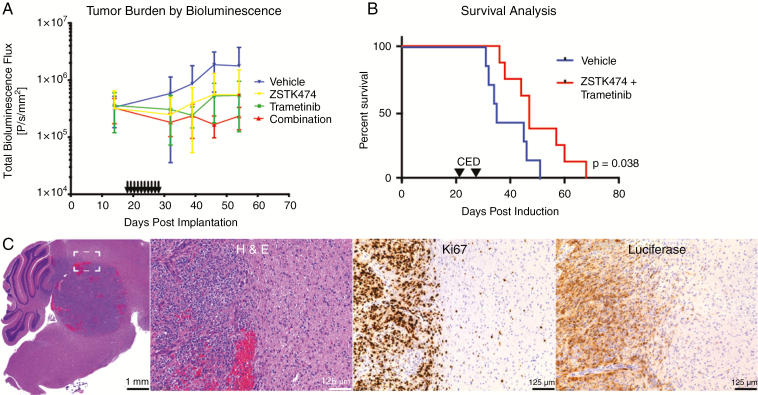
In vivo effects of PI3K and mitogen-activated protein kinase (MEK) inhibition. Quantification of pre- and posttreatment luciferase signal over time for subcutaneous xenograft experiments (A). Posttreatment time points were analyzed with two-way analysis of variance, with no significant difference between treatment groups (*F*(3,14) = 1.69, *P* = .21). Error bars SD. Kaplan–Meier curve of genetically engineered mouse model (GEMM) treated with either vehicle or combination of ZSTK474 and trametinib (B). Median survival time was 47 days for vehicle group versus 35 days for treatment group, log rank *P* = .038. Tumor histology from the intracranial GEMM showing features typical of diffuse intrinsic pontine glioma (DIPG), including markedly atypical cells with irregularly contoured nuclei, high cellular density, palisading necrosis, and infiltration of tumor cells into surrounding brain tissue. Scale bars as 1 mm and 125 µm, as indicated. (C).

A subcutaneous patient-derived DIPG mouse model was established to evaluate effects of combination treatment with ZSTK474 and trametinib versus each individual agent in vivo. Following tumor establishment and bioluminescence imaging (Supplementary Figure 2A), mice were treated via IP injection once daily, for 10 days with trametinib (3 mg/kg/day), ZSTK474 (400 mg/kg/day), or their combination. Quantification of total luciferase bioluminescent intensity in photons/second/mm^2^ was used as a measure of tumor volume (Supplementary Figure 2B). There was no difference in bioluminescent intensity for any treatment group over posttreatment measurements (two-way ANOVA, *F*(3,14) = 1.69, *P* = .21). However, there was a significant main effect for time *F*(3,14) = 5.04, *P* = .0045) and a trend for interaction effect (*F*(9,42) = 1.82, *P* = .09. (Figure 4A). Furthermore, numerous mice showed signs of treatment-related toxicity; however, none reached euthanasia endpoints.

A GEMM of DIPG, where tumor presence was confirmed by luciferase reporter imaging (Supplementary Figure 3A) and via histological staining (Supplementary Figure 3B and C) demonstrated a significant survival benefit for combination treatment using ZSTK474 and trametinib (*n* = 10) over vehicle (*n* = 11), with median survival 47 days versus 35 days, respectively (log rank *P* = .038) (Figure 4B). Histological analysis of harvested mouse brains demonstrated variable tumor growth characteristics with some samples showing predominantly intraventricular growth and others demonstrating intraparenchymal growth. Cytoarchitectural features characteristic of high-grade infiltrating astrocytoma were seen, including markedly atypical cells with irregularly contoured nuclei, high cellular density, palisading necrosis, and infiltration of tumor cells into surrounding brain tissue (appreciated at high magnification in Supplementary Figure 4A and B). In addition, tumor foci demonstrated markedly elevated Ki67 (a marker for proliferation) labeling indices. Luciferase staining confirmed RCAS transfection (Figure 4C).

### Neurotoxicity of ZSTK474 and Trametinib Delivered Via CED in Naïve Animals

**Fig. 5:**
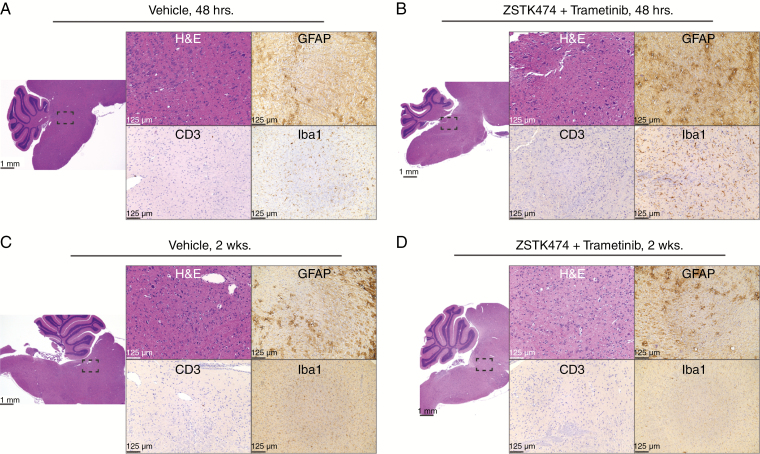
Safety profile of the convection-enhanced delivery (CED) of ZSTK474 and trametinib. Animals were Killed 48 hours after the last CED injection of either vehicle (A) or combination of both ZSTK474 and trametinib (B). No difference was observed in histology across treatments. Further animals were killed 2 weeks after the last CED injection of either vehicle (C) or combination of both ZSTK474 and trametinib (D). No difference was observed in histology across treatments. Scale bars as 1 mm and 125 µm, as indicated.

Treatment of naïve animals with CED of the combination therapy (ZSTK474 and trametinib) protocols used in the GEMM survival experiment revealed no acute or chronic features of inflammation or neurotoxicity beyond that observed in vehicle-treated animals Tissue was processed and stained with markers of gliosis (GFAP), T-cell lymphocytic infiltration (CD3), and microglia (Iba1) either 2 days (Figure 5A and B) or 2 weeks (Figure 5C and D) after the last CED. Histopathological analysis revealed reactive changes consistent with CED (needle tract with only minor associated focal hemorrhage, focal microcystic/edematous changes, reactive microgliosis and/or scant immune infiltration) in both combination and vehicle-treated animals, but no significant drug-specific neurotoxicity or neurological deficits were observed.

## Discussion

In this study, we demonstrated how dual PI3K and MEK inhibition synergistically inhibits DIPG cell growth in vitro while reducing tumor burden and prolonging survival in vivo. Although prior studies have demonstrated RTK and downstream PI3K/AKT/mTOR amplifications in DIPG, monotherapy against the PI3K pathway has failed to show a significant tumor response in various malignancies including DIPG and other CNS tumors.^7,8,12,13^ We have previously shown how combinatorial therapy resulted in increased caspase-dependent apoptosis.^10^ In this study, two CED injections were used to best approximate our clinical experience, where one or two CED injections are used in patients.^5^

Numerous pathways have been synergistically targeted along PI3K, such as histone deacetylase complex (HDAC),^29^ mitochondrial function,^30^ PIM kinase,^31^ and neuroligin.^32^ Of these, MEK inhibition has shown promise in preclinical work for dual therapy with PI3K inhibitors.^18,33^ Studies have shown how MEK signaling is often co-activated along PI3K in many types of cancers,^16,17^ including gliomas.^34^ In recent early-phase studies, two trials using PI3K inhibitors used in this study and one trial using PI3K/mTOR inhibitors PF-04691502 and gedatolisib, examined dual PI3K and MEK inhibition in advanced solid tumors.^16,35,36^ Partial responses were observed particularly in ovarian and endometrial KRAS mutant cancers, including 29% of ovarian cancers with BKM120 and trametinib.^35^ Moreover, frequent toxicity-related dose delays and reductions may have prevented sustained therapeutic drug levels across all trials. These issues emphasize not only potential problems in tolerating systemic dual PI3K and MEK treatment, but also a potential advantage of CED.

Novel PI3K inhibitors have varying BBB penetration and effects on PI3K isoforms, necessitating careful consideration when selecting agents for use against gliomas.^28,31,37,38^ Previous reports on selective PI3K inhibitors demonstrated rebound activation of non-inhibited PI3K isoforms, a possible mechanism behind resistance.^39,40^ Pan-PI3K inhibition may have theoretical advantages and disadvantages related to their antiangiogenic and immunomodulatory properties—while PI3K α and β isoforms are expressed in various tissues, δ and γ isoforms are largely expressed in leukocytes and affect neovascularization and immune signaling.^41^ However, reported pan-PI3K inhibitor adverse effects from clinical trials are more related to off-target effects (eg, PI3K-related kinases) and general dose-related toxicity than isoform-specific toxicity.^18^ ZSTK474 was chosen among novel PI3K inhibitors currently in phase I or II clinical trials (BKM120, BYL719, GDC-0032) because of both its profile as a pan-PI3K inhibitor and high oral maximum tolerated dose in mice.^27,28,39^ Immunoblot analysis of downstream effectors of PI3K/AKT and MEK/ERK pathways confirmed presence of mutual feedback inhibition and redundant signaling (Figure 3). Moreover, these results provide a mechanistic explanation for the synergistic effects of dual inhibition we observed. Though dual PI3K and MEK inhibition’s action on DIPG should not be reliant upon aberrant H3 function, H3 mutations in general and H3.3K27M in particular mutations cause widespread changes in gene expression. Owing to these widespread effects, we cannot rule out the influence of H3 mutations on the efficacy of concurrent PI3K and MEK inhibition.^42^ PI3K signaling has been shown to be aberrantly activated in many pediatric CNS neoplasms irrespective of H3K27 mutation status and agents targeting PI3K signaling are in clinical trials for pediatric brain tumors of various etiologies.^43^

Ultimately, a more comprehensive multiple dosing regimen coupled with pharmacokinetics/pharmacodynamics studies will be required for correlating in vitro synergistic effects with effects in animal models. Although our GEMM experiments demonstrated prolonged survival with PI3K and MEK treatment via CED, our subcutaneous xenograft studies showed no significant difference between treatment groups. However, there was a trend toward an interaction with the other main effect of time. Differences in tumor volume over time dependent on treatment group would suggest that treatment did affect tumor growth. Owing to decreased number of animals in the trametinib group secondary to poor luciferase signal (*n* = 3), our experiments were not adequately powered for the level of variability we observed between individual animals’ tumor volumes. The numerous side effects we observed ultimately convinced us to use a direct drug delivery approach instead. Nevertheless, prior studies, our current in vitro data, and our GEMM in vivo results present a strong rationale for dual PI3K and MEK inhibitor therapy for cancer treatment and is thus being exploited in the clinic at the early-phase clinical trial level in other malignancies.^44^

Moreover, trametinib has been reported to have poor BBB penetration; as such, CED was used to maximize intratumoral concentration.^45,46^ Though more invasive than systemic administration, CED allows for the entire tumor to be permeated by the drug given. Further, it minimizes the drug needed at therapeutic concentrations, thus limiting the development of systemic toxicities.^47^ Of note, no clear systemic toxicity nor neurotoxicity was observed when trametinib and ZSTK474 were given via CED; significant systemic toxicity (weight loss, food avoidance) was instead observed when the drugs were given via systemic routes, consistently with clinical evidence suggesting that the development of dose-limiting toxicities for these agents is common and potentially increased by their combination.^48,49^ These findings are consistent with the results of both preclinical work and clinical trial results demonstrating the safety of CED in DIPG patients, and emphasize the strengths of a local delivery approach in the treatment of brain malignancies.^4,5,50^

## Funding

This research was supported in part by Cristian Rivera Foundation, McKenna Claire Foundation, The Lyonhearted Foundation, Christian Koehler Foundation, Brooke Healey Foundation, Fly a Kite Foundation, Children’s Brain Tumor Family Foundation, Joshua’s Wish, Lily LaRue Foundation; and Alex Lemonade Stand Foundation’s Pediatric Oncology Student Training Grant (U.T.); and American Brain Tumor Association Medical Student Summer Fellowship in honor of Collegiate Charities Dropping the Puck on Cancer and Super Lucy (R.C.).

## Supplementary Material

vdz004_suppl_Supplementary_Figures

vdz004_suppl_Supplementary_Figures_Legends
