# Supplementary material for: Combined targeting of PI3K and MEK effector pathways via CED for DIPG therapy
Source: Neurooncol Adv. 2019 May 28;1(1):vdz004. doi: 10.1093/noajnl/vdz004 (PMC7212917; doi:10.1093/noajnl/vdz004)
Supplement: vdz004_suppl_Supplementary_Figures [file vdz004_suppl_supplementary_figures.pdf]

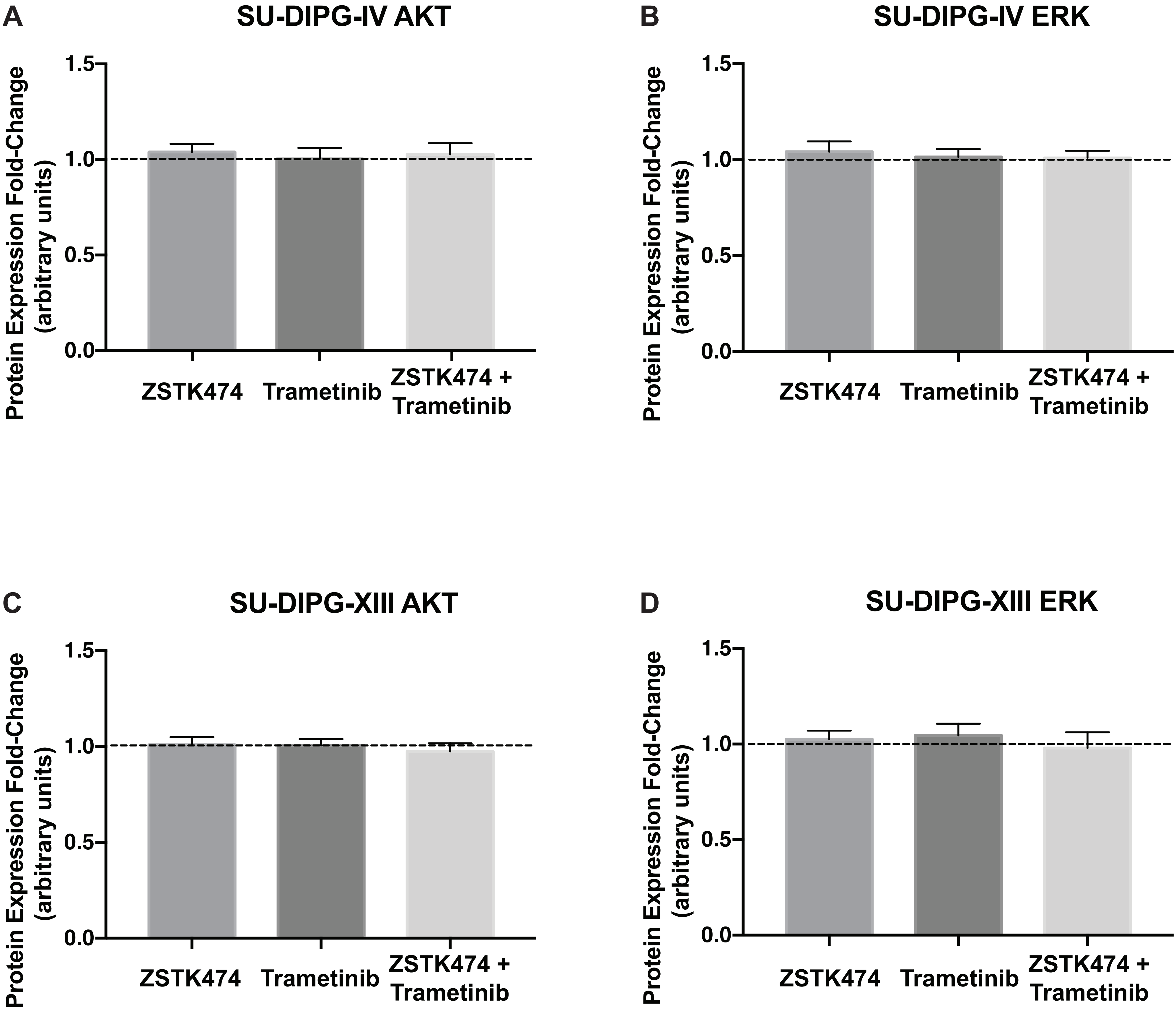

Supplementary Figure 1

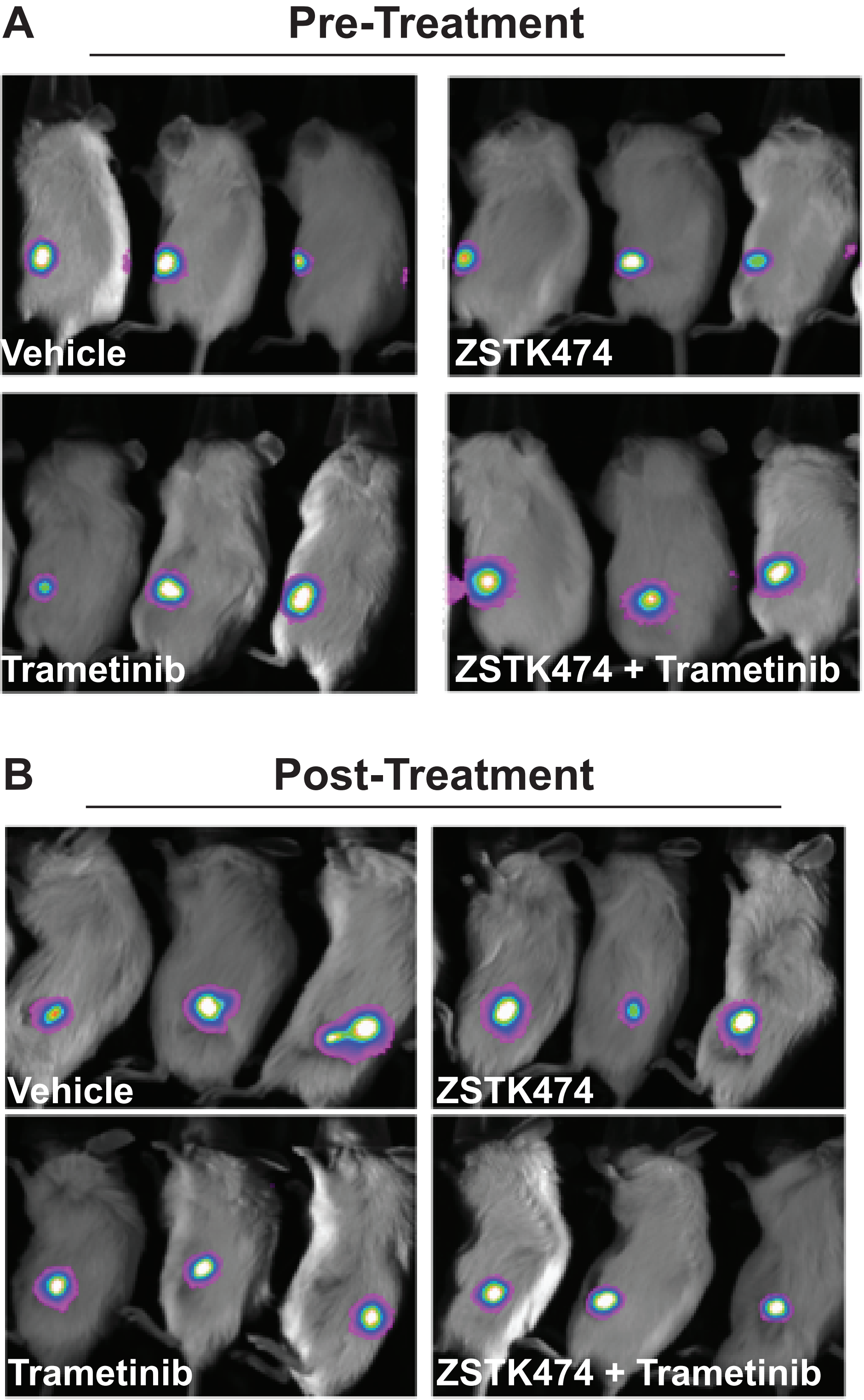

Supplementary Figure 2

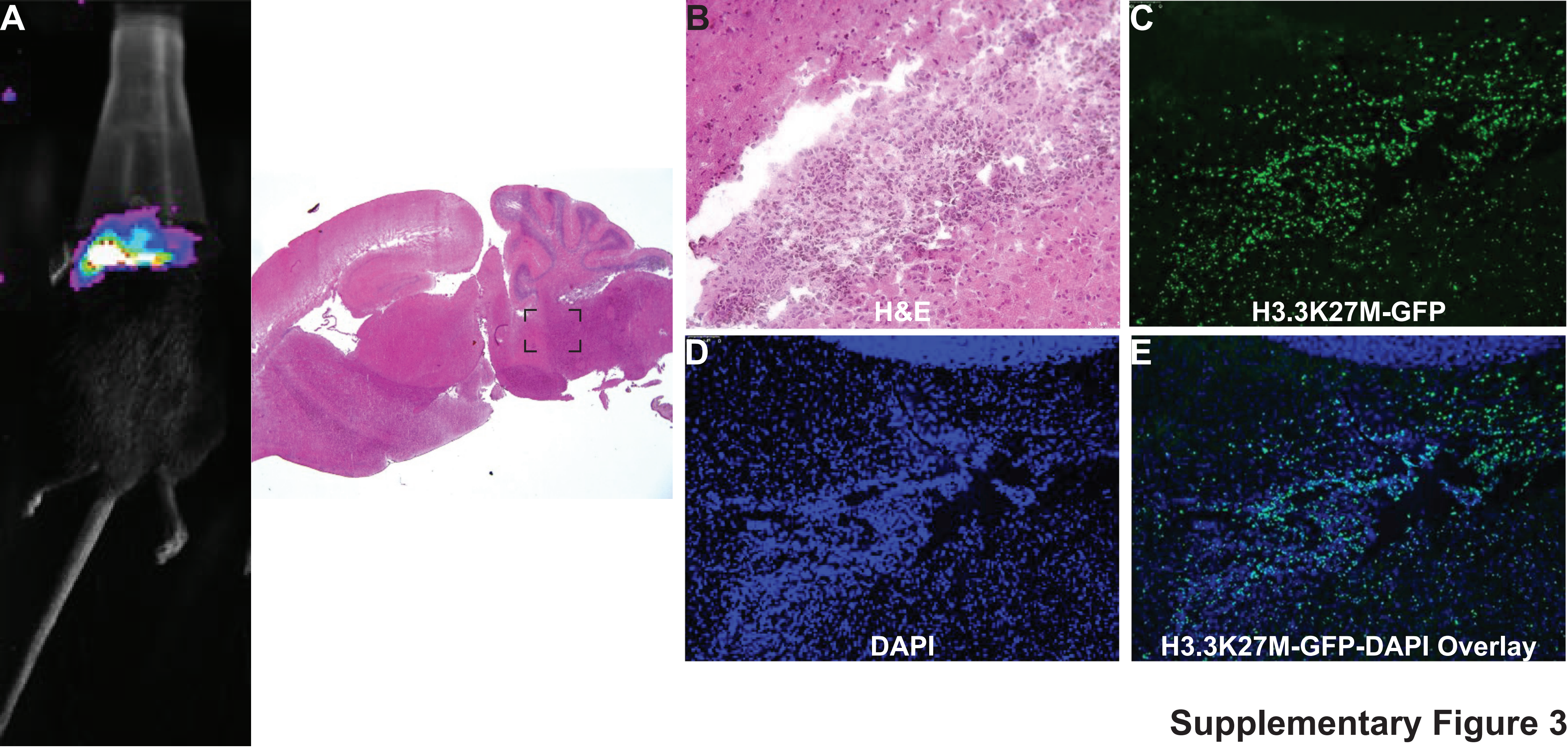

Supplementary Figure 3

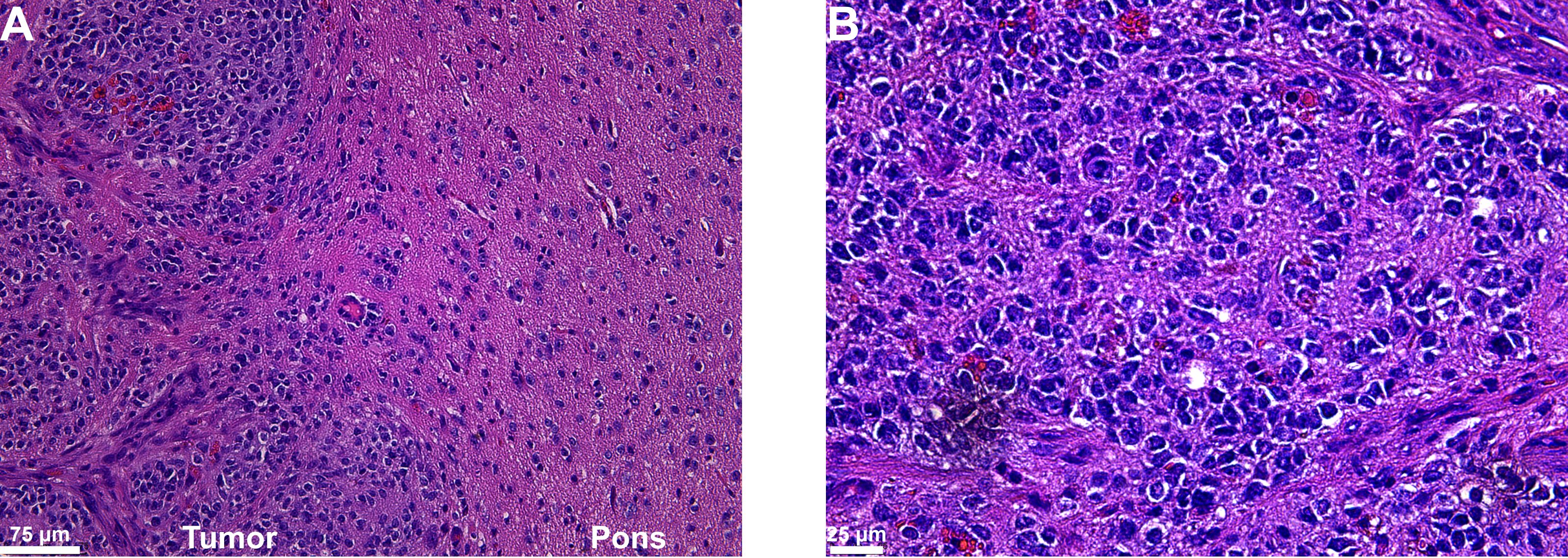

Supplementary Figure 4
