# Supplementary material for: Combined targeting of PI3K and MEK effector pathways via CED for DIPG therapy
Source: Neurooncol Adv. 2019 May 28;1(1):vdz004. doi: 10.1093/noajnl/vdz004 (PMC7212917; doi:10.1093/noajnl/vdz004)
Supplement: vdz004_suppl_Supplementary_Figures_Legends [file vdz004_suppl_supplementary_figures_legends.docx]

**Supplementary Table and Figure Legends to “Combined Targeting of PI3K and MEK Effector Pathways via CED for DIPG Therapy.”**

**Supplementary Figure 1:** Effects of PI3K and MEK Inhibition on total levels of AKT and ERK. Semi-quantification of AKT (A) and ERK (B) as protein-expression fold-change as a result of treatments, as compared to 0.1% DMSO vehicle. in SU-DIPG-IV cells. Semi-quantification of AKT (C) and ERK (D) as protein-expression fold-change as a result of treatments, as compared to 0.1% DMSO vehicle. in SU-DIPG-XIII cells.

**Supplementary Figure 2:** Luciferin imaging in a subcutaneous xenograft mouse model of DIPG. Luciferin imaging was carried out before (A) and after (B) treatment.

**Supplementary Figure 3**: Histological analysis of brain tissue in a GEMM of DIPG confirms transfection. Luciferase scanning confirms tumor presence (A). Histological assessment of extracted brain tissue showing H&E characteristic of tumor tissue (B), transfection with H3.3K27M-GFP (C), and the overlay of this staining with DAPI (D) (overlay in E).

**Supplementary Figure 4**: High magnification histological analysis of brain tissue in a GEMM of DIPG (same sample as figure 4) shows a DIPG-specific phenotype. 200x magnification where the tumor-brain border can be observed (A). 400x magnification where irregularly contoured nuclei and high cellular density can be appreciated (B). Scale bars as 75 µm and 25 µm, as indicated
